# An interactive videogame designed to optimize respiratory navigator efficiency in children undergoing cardiac magnetic resonance

**DOI:** 10.1186/1532-429X-18-S1-O10

**Published:** 2016-01-27

**Authors:** Sean M Hamlet, Jonathan D Suever, Jonathan D Grabau, Gregory J Wehner, Moriel Vandsburger, Kristin N Andres, David Powell, Vincent L Sorrell, Brandon K Fornwalt

**Affiliations:** 1 grid.266539.d0000000419368438Electrical and Computer Engineering, University of Kentucky, Lexington, KY USA; 2 grid.266539.d0000000419368438Pediatrics, University of Kentucky, Lexington, KY USA; 3 grid.280776.c0000000403941447Institute for Advanced Application, Geisinger Health System, Danville, PA USA; 4 grid.266539.d0000000419368438Biomedical Engineering, University of Kentucky, Lexington, KY USA; 5 grid.266539.d0000000419368438Physiology, University of Kentucky, Lexington, KY USA; 6 grid.266539.d0000000419368438Medicine, University of Kentucky, Lexington, KY USA; 7 grid.266539.d0000000419368438Cardiology, University of Kentucky, Lexington, KY USA

## Background

Advanced cardiac magnetic resonance (CMR) acquisitions often require long scan durations that necessitate respiratory navigator gating. This is particularly important in children with limited ability to hold their breath. We hypothesized that visual feedback of diaphragm position using an interactive videogame during CMR would increase navigator efficiency and improve image quality in children.

## Methods

**Figure 1:**
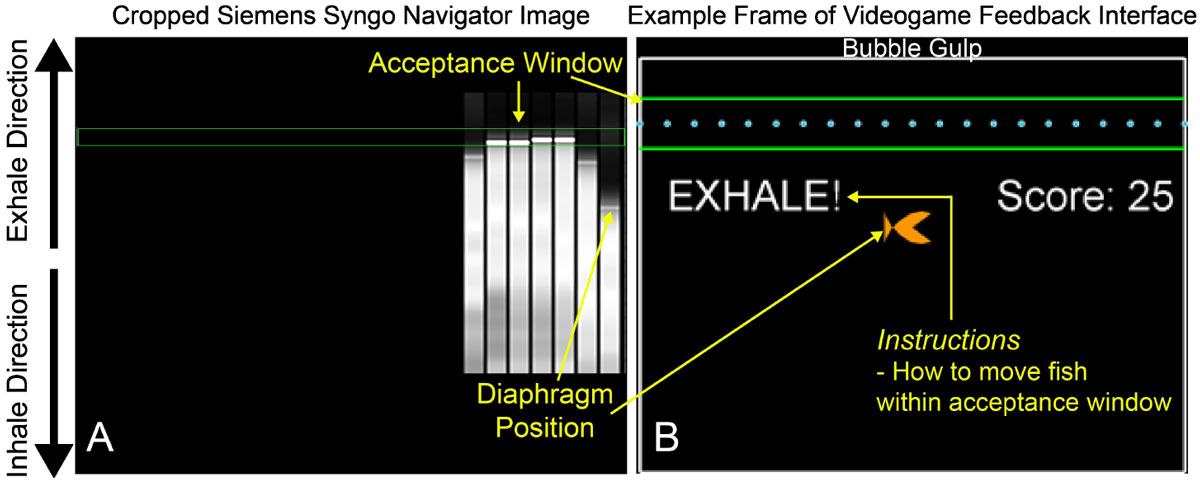
**(A) Cropped version of the Siemens Syngo navigator image that was processed in real-time during CMR acquisition to yield the feedback game**. (B) Example frame of the navigator feedback videogame interface, which is shown to the child during DENSE CMR (yellow overlay text is not shown to the child). The fish's position is restricted to vertical translation and represents the diaphragm position relative to the acceptance window location (green lines). The objective of the game is to control the fish so that the fish "gulps" as many bubbles as possible, which scroll across the screen from right to left in between the green lines (acceptance window). A score counter incentivizes children to spend more time with their diaphragm within the acceptance window.

A feedback videogame was developed using MATLAB. The navigator image provided within the Siemens Syngo user-interface (Figure 1A) was processed in real-time to yield a kid-friendly representation of diaphragm position which was then projected to the subject in the scanner (Figure 1B). The game used a point-based system to incentivize children to hold their diaphragm within the navigator acceptance window (± 3 mm) throughout image acquisition (Figure 1).

Using a 3T Siemens Tim Trio, 20 healthy children (Age: 13 ± 3, 35% female) underwent a navigator-gated 2D spiral cine displacement encoding with stimulated echoes (DENSE) acquisition (mid-ventricular, basal, apical, and 4-chamber images) first with no feedback and then with the videogame. Additional imaging parameters were: 12 spiral interleaves, voxel size: 2 × 2 × 8 mm, TE/TR: 1.08/17, flip angle: 20°, 1 average. Between the acquisitions with no feedback and those with the videogame, each child underwent two 30-heartbeat practice scans to familiarize themselves with the videogame. Navigator efficiency and signal-to-noise ratio (SNR) were determined for each subject and compared using a paired student's t-test.

## Results

**Figure 2:**
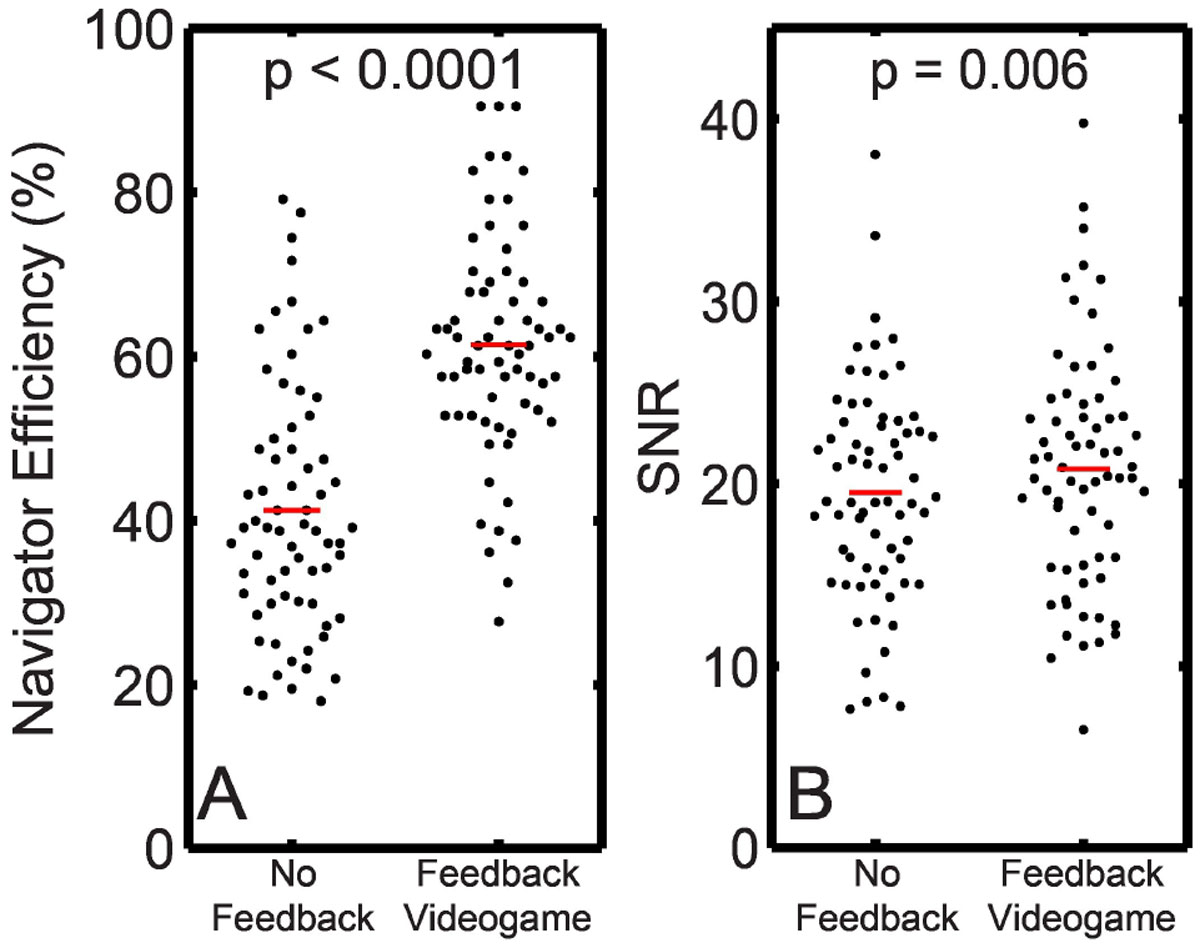
**Use of a navigator videogame feedback system improves both navigator efficiency (A) and SNR (B) of DENSE images acquired in children**.

The videogame improved navigator efficiency by 50% (p < 0.0001, Figure 2A) and improved SNR by 7% compared to scans without feedback (p = 0.006, Figure 2B).

## Conclusions

Use of a diaphragmatic feedback videogame during navigator-gated DENSE CMR can improve navigator efficiency by 50% in children. The videogame also has a slight positive effect on image quality with a 7% increase in SNR, potentially due to the shorter scan durations leading to reduced heart rate variability. These findings should be generalizable to all CMR acquisition sequences which utilize a respiratory navigator.

